# Importance of Preoperative Screening Strategies for Coronavirus Disease 2019 in Patients Undergoing Cesarean Sections: A Retrospective, Large Single-Center, Observational Cohort Study

**DOI:** 10.3390/jcm10040885

**Published:** 2021-02-22

**Authors:** Ha-Jung Kim, Hyun-Seok Cho, Mi-Young Lee, Hyungtae Kim, Woo-Jong Choi, Hye-Sung Won, Young-Jin Ro, In-Cheol Choi

**Affiliations:** 1 Department of Anesthesiology and Pain Medicine, Asan Medical Center, University of Ulsan College of Medicine, Seoul 05505, Korea; alexakim06@gmail.com (H.-J.K.); s2iichiro@gmail.com (H.-S.C.); yjro@amc.seoul.kr (Y.-J.R.); icchoi@amc.seoul.kr (I.-C.C.); 2 Department of Obstetrics and Gynecology, Asan Medical Center, University of Ulsan College of Medicine, Seoul 05505, Korea; poptwinkle@hanmail.net

**Keywords:** COVID-19, cesarean section, screening strategy, RT–PCR

## Abstract

During the coronavirus disease 2019 (COVID-19) pandemic, many guidelines have recommended postponing non-emergency operations. However, cesarean sections cannot be indefinitely delayed. Our institution has established a COVID-19 screening strategy for patients undergoing cesarean section. We evaluated the usefulness of this screening strategy. Parturients undergoing cesarean section at our center during the first peak of the COVID-19 outbreak were retrospectively analyzed. Each parturient underwent a questionnaire survey evaluating epidemiological correlation and symptoms at admission. Reverse transcriptase–polymerase chain reaction (RT–PCR) testing and/or chest radiography were performed. In total, 296 parturients underwent cesarean section. All elective and 37 emergency cesarean sections were performed in general operating rooms because they were considered to have a low risk of COVID-19 infection through the screening process. However, 42 emergency cases were performed in negative-pressure operating rooms with full personal protective equipment (PPE) because the RT–PCR results could not be confirmed in a timely manner. None of them were positive for RT–PCR, and there were no cases of nosocomial infection. A comprehensive preoperative screening strategy, including symptomatic and epidemiological correlation, PCR, and/or imaging test, should be performed in patients undergoing cesarian section. Further, cesarean sections in parturients with unconfirmed COVID-19 status should be performed in a negative-pressure operating room with appropriate PPE.

## 1. Introduction

Towards the end of August 2020, approximately 25 million people worldwide had been infected with severe acute respiratory syndrome coronavirus 2 (SARS-CoV-2), and 850,000 patients had died in association with coronavirus disease 2019 (COVID-19). Therefore, COVID-19 is currently the most interesting medical issue globally. In December 2019, this new strain of coronavirus was first reported in Wuhan City, Hubei Province, China [1]. Since then, SARS-CoV-2 infection has rapidly spread worldwide, and on 11 March 2020, the World Health Organization declared a global pandemic. Many countries have made enormous efforts to minimize the spread of COVID-19, including closing their national borders to all nonresidents and implementing mandatory quarantine measures. In addition, medical experts have made tremendous efforts to develop a medicine for treatment and a vaccine for preventing the transmission of the disease. Despite these efforts, there is still no effective treatment for COVID-19 or vaccine against SARS-CoV-2, a type of RNA virus with error-prone RNA-dependent RNA polymerases and showing frequent mutations [2]. Because it is difficult to control, COVID-19 has unprecedentedly spread worldwide, and a new phase of the pandemic has begun.

South Korea, which is geographically close to and actively trading with China, is one of the countries that experienced a relatively quick start of the coronavirus epidemic. On 20 January 2020, the first diagnosed case of COVID-19 was reported in Korea, and by 17 February, an explosive spread of the disease occurred among members of a certain religion who attended a worship service. Therefore, the Korean government decided to raise the alert level for COVID-19 from orange to red, which is the highest, on 23 February 2020 [3]. Subsequently, the rate of COVID-19 spread relatively decreased; however, locally transmitted and imported cases from other countries steadily continued to occur.

During this pandemic, many guidelines recommended reducing unnecessary visits to hospitals and postponing some non-emergency operations [4]. However, cesarean sections, whether emergency or elective, cannot be indefinitely delayed. Especially in cases of fetal distress requiring emergency surgery, a reduced decision-to-delivery time has been known to improve the outcome [5]. Nevertheless, a prescreening process for COVID-19 before a cesarean section is required for the safety of the parturient herself, the neonate, other patients admitted in the hospital, and medical workers. However, no guideline has recommended an appropriate screening process before a cesarean section. Previous studies on pregnancy and COVID-19 were based on information from pregnant women with COVID-19 pneumonia. However, limited data are available on pregnant women scheduled to undergo cesarean sections in this pandemic situation. Therefore, our institution has established and applied our own COVID-19 screening strategy for parturients undergoing cesarean section. This study aimed to evaluate the usefulness of preoperative screening strategies in preventing COVID-19 spread in patients undergoing cesarean section.

## 2. Materials and Methods

This retrospective study was approved by the Institutional Review Board of Asan Medical Center (protocol no. 2020-1126), and the requirement for written informed consent was waived owing to the retrospective nature of this study. This study was conducted following the rules of the Declaration of Helsinki.

### 2.1. Study Population

We retrospectively reviewed the electronic medical records of all patients who underwent elective or emergency cesarean section between 23 February 2020 and 5 May 2020 at our institution, a tertiary center in Seoul, Korea. The study period covered the first peak of the COVID-19 outbreak in Korea. We started implementing our preoperative screening strategy for COVID-19 in patients undergoing cesarean section on 23 February 2020.

### 2.2. Data Collection

All data were obtained from the electronic medical record system of our hospital. We investigated the demographic data, medical and social history, reverse transcriptase-polymerase chain reaction (RT–PCR) results, chest radiographic findings, surgical data, and anesthetic data of all parturients. In addition, we also collected the data of neonates and the postoperative follow-up data of parturients suspected to have COVID-19. The demographic data included age, height, weight, the American Society of Anesthesiologists Physical Status classification, and gestational age. Medical and social history included signs and symptoms such as fever/chills, respiratory symptoms, myalgia, new loss of taste or smell, exposure to patients with COVID-19, and travel history. Surgical and anesthetic data included diagnosis, surgery type (elective or emergency), anesthesia and operation times, operating room type, use of personal protective equipment (PPE), and anesthetic method. The data of neonates were also collected, which included body weight, Apgar scores at 1 and 5 min, application of negative-pressure isolation, and admission to the neonatal intensive care unit.

### 2.3. Preoperative SARS-CoV-2 Screening Process

**Figure 1:**
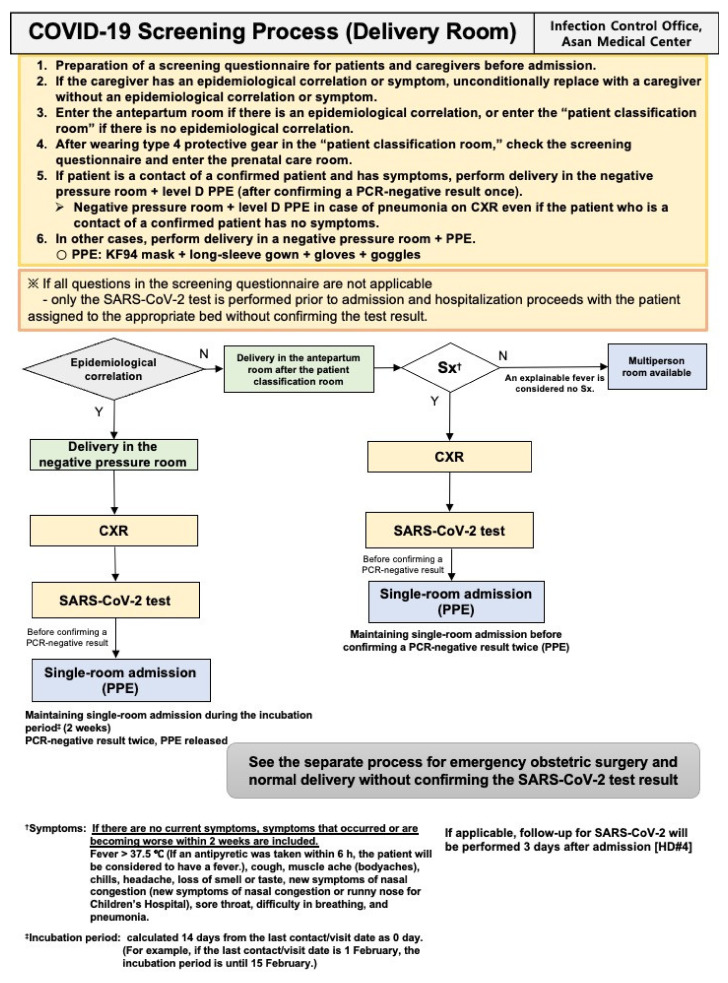
COVID-19 screening process for patients admitted to delivery rooms. COVID-19: coronavirus disease 2019; CXR: chest X-ray; HD: hospital day; PCR: polymerase chain reaction; PPE: personal protective equipment; SARS-CoV-2: severe acute respiratory syndrome coronavirus 2; Sx: symptom.

**Figure 2:**
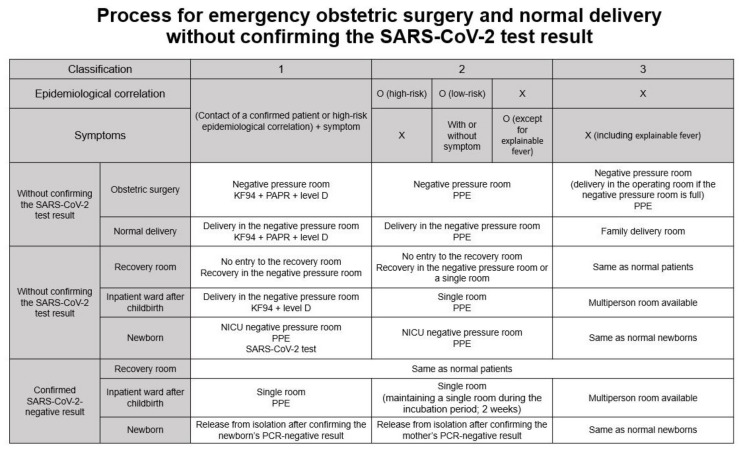
Protocol for emergency obstetric surgery and normal delivery without confirmation of SARS-CoV-2 test result. PPE comprises a KF94 mask, long-sleeve gown, gloves, and goggles. See Figure 3 for the types of PPE used at the Asan Medical Center (AMC). PAPR: powered air-purifying respirator; PPE: personal protective equipment (KF94 mask + long-sleeve gown + gloves + goggles); SARS-CoV-2: severe acute respiratory syndrome coronavirus 2; NICU: neonatal intensive care unit.

**Figure 3:**
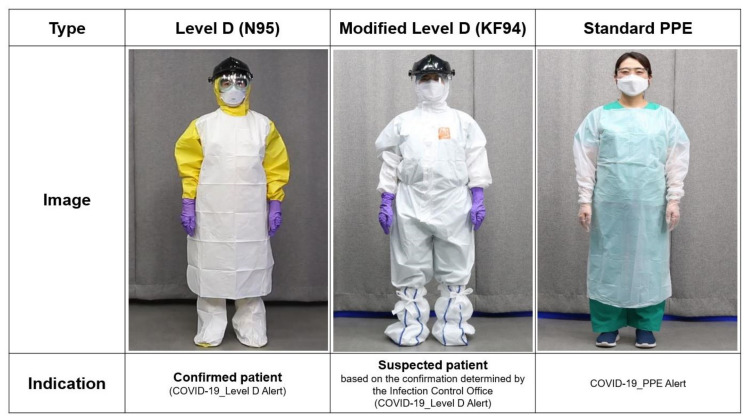
Types of PPE used at AMC. PPE: personal protective equipment; AMC: Asan Medical Center; COVID-19: coronavirus disease 2019.

All patients visiting our hospital were evaluated according to a screening strategy. Several prescreening processes were available depending on each patient’s situation. For delivery cases, before admission, each parturient and her caregiver were requested to respond to our detailed questionnaire asking for signs or symptoms of COVID-19 (Supplementary Figure S1). The questionnaire was based on national and global pandemic information and aimed at evaluating the epidemiological correlation with COVID-19. Considering the new outbreak in Korea and abroad, the questionnaire was daily updated [6]. The room for admission was assigned on the basis of the questionnaire results. Thereafter, a PCR test with or without chest X-ray examination was performed (Figure 1). In brief, the principle was that cesarean section should be performed after a SARS-CoV-2 test with or without chest radiographic results. However, if an emergency cesarean section was necessary before the results were available, the process presented in Figure 2 was applied. The operating room for cesarean section and the type of PPEs (Figure 3) were determined depending on epidemiological correlation and symptoms. The recovery room and inpatient ward for the parturients and the admission room for the newborns were also decided following our protocol.

### 2.4. Statistical Analysis

Categorical variables are presented as numbers (%), and continuous variables are expressed as mean (standard deviation) or median (interquartile range).

## 3. Results

**Figure 4:**
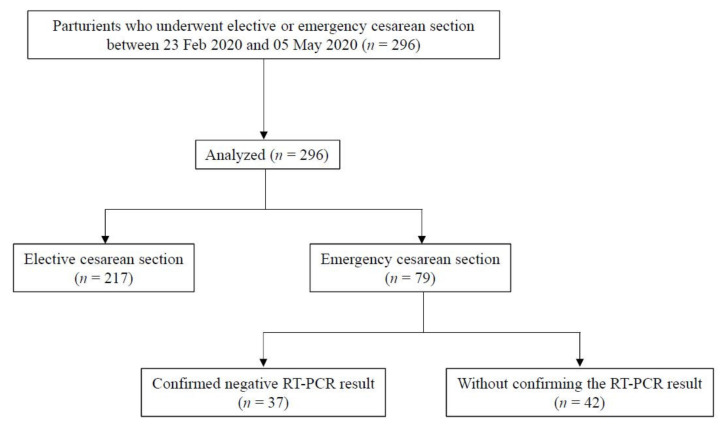
Flow diagram for analysis of patients.

A total of 296 parturients, who underwent cesarean section during the 72 days defined as the first peak of the COVID-19 outbreak in South Korea, were enrolled in the analysis (Table 1, Figure 4). The COVID-19-related factors of the patients are presented in Table 2. Among the 296 parturients, 217 underwent elective cesarean section. The most common cause of elective surgeries was a previous cesarean section, followed by cephalopelvic disproportion and multifetal pregnancies. All parturients who underwent elective cesarean section did not have any epidemiological correlation. Only one parturient reported fever with chills, whereas one other parturient reported having a cough on the questionnaire. Seven parturients undergoing elective cesarean section repeated the PCR test owing to newly developed fever after the admission. However, all preoperative PCR results were negative, and the surgeries were performed in a regular operating room. Most of the parturients received neuraxial anesthesia, including combined spinal-epidural anesthesia and simple spinal anesthesia.

The other 79 cases were emergency cesarean sections caused by failed induction of labor, cephalopelvic disproportion, and other reasons. In the emergency group, 4 parturients had an epidemiological correlation, and 11 parturients reported having some of the symptoms on the questionnaire. In 37 cases, fortunately, we had time to wait for the results, and the surgeries were performed after the confirmation of a negative PCR result. In contrast, surgeries were performed in a negative-pressure operating room, with the medical staff wearing PPE, in 42 cases for which we could not wait for the PCR results. Of these, one parturient with epidemiological correlation, fever, and myalgia belonged to class 1; 3 parturients with fever and/or respiratory symptom belonged to class 2, and 38 parturients without any sign or symptom belonged to class 3 (Figure 2). Fortunately, all patients who underwent cesarean section without PCR results in this study eventually received negative RT–PCR results and were released from quarantine. Approximately 85% of emergency cases were performed under neuraxial anesthesia.

The characteristics of neonates born in the study period are presented in Table 3. Because four babies whose mothers belonged to classes 1 and 2 needed to be isolated from other neonates, they were transferred to the negative-pressure room in the neonatal intensive care unit. Table 4 shows the outcomes of the neonates isolated in the negative-pressure room according to our protocol.

## 4. Discussion

This study demonstrated that an appropriate screening strategy for COVID-19 in patients undergoing cesarean section protects the parturients, neonates, and healthcare providers against exposure to SARS-CoV-2, even during the COVID-19 pandemic. This strategy starts with a thorough questionnaire survey assessing symptoms such as fever, cough, rhinorrhea, and newly developed loss of smell or taste and evaluating whether a patient has a potential epidemiological correlation with COVID-19. Moreover, the confirmation of RT–PCR results should be included as a diagnostic tool if time and resources allow it. With this strategy, we experienced no excessive postponement of cesarean section cases, and we were able to protect all the patients and medical workers at our institution.

SARS-CoV-2 is mainly transmitted via respiratory droplets and secretions containing the virus. It is a type of coronavirus similar to the SARS-CoV and Middle East respiratory syndrome coronavirus (MERS-CoV). Although SARS-CoV-2 has a lower fatality rate (2.3%) than SARS-CoV (9.5%) and MERS-CoV, the reproductive number of SARS-CoV-2 seems to be higher than those of SARS (1.7–1.9) and MERS (< 1) [7]. Moreover, some individuals infected with SARS-CoV-2 may be asymptomatic, and the virus has high infectivity even in the incubation period [8,9]. Based on these characteristics of SARS-CoV-2, COVID-19 is likely to be more easily transmitted in the community. Therefore, an appropriate screening process before admission and surgery is essential for preventing the spread of COVID-19 in a medical institution.

The screening process requires several steps. The evaluation of epidemiological correlation was the first step in our screening process. As community transmission is common in COVID-19, this step was a definite requirement. The next step was the assessment of clinical manifestations. Patients with COVID-19 show various clinical manifestations, including fever, cough, sore throat, nasal congestion, dyspnea, fatigue, myalgia, gastrointestinal symptoms, and new loss of taste or smell [10,11]. Thus, a parturient with even a single symptom was assumed to be a COVID-19 patient and was separated from others. Thereafter, RT–PCR test and chest X-ray examination were performed for a differential diagnosis. Although RT–PCR assay has some limitations, including the potential of false-negative results and changes in accuracy over the disease course, it is widely considered the standard diagnostic tool to confirm COVID-19 [12,13,14]. In this study, RT–PCR was performed in all parturients who underwent cesarean section. The preoperative RT–PCR results were negative in elective cesarean section cases. Moreover, the RT–PCR results were negative in 33 unexpected emergency cesarean sections after the failure of normal delivery.

The RT–PCR assay takes several hours to complete, and some problems in the PCR kit supply may occur. Performing cesarean section after confirming a negative RT–PCR result could be the safest way to prevent healthcare providers from being exposed to SARS-CoV-2. However, there are some immediate cases in which waiting for the test results is impossible. For such cases, we recommend performing the surgery in a negative-pressure operating room with the medical staff wearing PPE. In our study, parturients without a confirmed SARS-CoV-2 result underwent cesarean section in the negative-pressure operating room, and they all presented with negative RT–PCR results after the surgery. Our strategy includes a few steps to identify some parturients with suspected COVID-19 even before an RT–PCR test, which may be effective. However, using a negative-pressure operating room for emergency cesarean sections in patients without PCR results seemed to reduce the risk of COVID-19 spread in entire regular operating rooms.

Some patients who had newly developed fever or other respiratory symptoms repeated the RT–PCR test before the cesarean section. A single RT–PCR test for SARS-CoV-2 costs $70–100 in Korea, and our institution paid for the test fees. During the 75 days covered by this study, our hospital paid approximately $30,000 for the tests in all parturients. Performing RT–PCR tests in all preoperative patients posed an economic burden to our institution and seemed to be an excessive strategy. However, this cost was much lower than the expected loss of $12,500,000 from contamination of the operating room by SARS-CoV-2, which would have involved closing the entire operating rooms of our hospital for 2 weeks. In addition, this strategy could protect our medical staff working in operating rooms and other patients undergoing surgery.

Knowledge about coronavirus infections that occur during pregnancy is still limited. There is currently no evidence for intrauterine infection by vertical transmission in parturients with COVID-19 pneumonia. Several papers reported that neonates born to mothers with COVID-19 were not infected, and the amniotic fluid, cord blood, and breastmilk showed negative SARS-CoV-2 results [15,16,17]. On the contrary, some articles demonstrated the possibility of vertical transmission based on positive nasopharyngeal swab test results of neonates from mothers with COVID-19 [18,19]. However, all previous studies were based on data from small groups of patients, and the potential of vertical transmission of SARS-CoV-2 remains unclear. Even if the vertical transmission does not occur, the neonate is still likely to be exposed upon birth to the mother’s droplets and secretions containing the virus. Thus, the medical staff should be prepared to appropriately manage neonates with a risk of COVID-19 infection. Our institution has a strategy for dealing with neonates born from mothers with confirmed or suspected COVID-19, and we transferred four babies to the negative-pressure room according to our strategy. Screening parturients for COVID-19 might help guide the management of newborns and prevent the nosocomial spread of COVID-19 in neonates.

Our report had several limitations. First, the risk of COVID-19 transmission in the community in South Korea was relatively lower than in other countries, as mask-wearing and social distancing were strongly enforced by the Korean government and individuals suspected to be exposed to SARS-CoV-2 were thoroughly isolated even during the peak of the COVID-19 outbreak. Second, the current screening strategy is labor-intensive and requires abundant medical resources. Our hospital, which has 2715 beds, is the largest medical center in Korea. It is well equipped and has sufficient manpower. Ideally, the PCR test should be performed in all parturients; however, this could be difficult to implement in reality. Thus, we emphasize that a customized screening strategy based on the available medical resources in each institution should be established.

## 5. Conclusions

In summary, in pregnant women undergoing cesarean section, careful evaluation of clinical symptoms and epidemiological correlation, PCR test, and imaging tests should be performed and repeated if necessary. Moreover, cesarean sections in parturients with suspected COVID-19 should be conducted in a negative-pressure operating room with appropriate PPE. We have maintained our medical service for pregnant women using the aforementioned systematic preoperative COVID-19 screening strategy, and this strategy is still daily updated. Through such efforts, we were able to maintain the usual level of delivery and care for a high volume of parturients. Although the PCR test is the most important tool for diagnosis, an optimal screening strategy without needing PCR results could reduce the risk of COVID-19 spread from parturients. Our report may be helpful in planning the management of parturients undergoing cesarean section, particularly if COVID-19 remains in a pandemic status.

## Figures and Tables

**Table 1:** Characteristics of the parturients.

	Emergency (*n* = 79)	Elective (*n* = 217)	Total (*n* = 296)
Age, years (SD)	34 (4)	36 (4)	35 (4)
BMI, kg/m^2^ (IQR)	26.4 (24.6–28.3)	26.7 (24.4–28.8)	26.6 (24.4–28.7)
Gestational age, days (SD)	254 (28)	266 (11)	263 (18)
Diagnosis, *n* (%)			
Previous cesarean section	7 (8.9%)	53 (24.4%)	60 (20.3%)
Cephalopelvic disproportion	11 (13.9%)	37 (17.1%)	48 (16.2%)
Multifetal pregnancy	6 (7.6%)	36 (16.6%)	42 (14.2%)
Known fetal anomaly	7 (8.9%)	19 (8.8%)	26 (8.8%)
Placenta previa	3 (3.8%)	20 (9.2%)	23 (7.8%)
Malpresentation	5 (6.3%)	13 (6.0%)	18 (6.1%)
Failed induction of labor	16 (20.3%)	1 (0.5%)	17 (5.7%)
Fetal distress	6 (7.6%)	2 (0.9%)	8 (2.7%)
Preeclampsia	4 (5.1%)	4 (1.8%)	8 (2.7%)
Anesthetic methods, *n* (%)			
GA	12 (15.2%)	8 (3.7%)	20 (6.8%)
SA	22 (27.8%)	13 (6.0%)	35 (11.8%)
CSEA	45 (57.0%)	196 (90.3%)	241 (81.4%)
ASA classification, *n* (%)			
2	73 (92.4%)	212 (97.7%)	285 (96.3%)
3	6 (7.6%)	5 (2.3%)	11 (3.7%)
Duration of anesthesia, min (SD)	63.6 (11.9)	68.8 (25.9)	67.4 (23.1)
Duration of operation, min (SD)	41.8 (10.0)	47.0 (41.2)	45.6 (35.7)

Data are presented as *n* (%), mean (SD), or median (IQR). SD: standard deviation; IQR: interquartile range; BMI: body mass index; GA: general anesthesia; SA: spinal anesthesia; CSEA: combined spinal-epidural anesthesia; ASA: American Society of Anesthesiologists.

**Table 2:** COVID-19-related factors of patients undergoing cesarean section.

		Emergency (*n* = 79)	Elective (*n* = 217)	Total (*n* = 296)
Epidemiological correlation, *n* (%)		4 (5.1%)	0	4 (1.4%)
Contact with a confirmed patient, *n* (%)		0	0	0
Symptoms on the questionnaire, *n* (%)				
	Fever	10 (12.7%)	1 (0.5%)	11 (3.7%)
	Cough	1 (1.3%)	1 (0.5%)	2 (0.7%)
	Dyspnea	0	0	0
	Chills	0	1 (0.5%)	1 (0.3%)
	Myalgia	1 (1.3%)	0	1 (0.3%)
	Sore throat	0	0	0
	New loss of taste or smell	0	0	0
Body temperature, *n* (%)				
Day before the cesarean section	< 37.5 °C	41 (51.9%)	206 (94.9%)	247 (83.4%)
	≥ 37.5 °C	6 (7.6%)	2 (0.9%)	8 (2.7%)
Morning of the operation day	< 37.5 °C	72 (91.1%)	190 (87.6%)	262 (88.5%)
	≥ 37.5 °C	4 (5.1%)	0	4 (1.4%)
Before entering the operating room	< 37.5 °C	47 (59.5%)	107 (49.3%)	154 (52.0%)
	≥ 37.5 °C	10 (12.7%)	0	10 (3.4%)
Before anesthesia	< 37.5 °C	70 (88.6%)	203 (93.5%)	273 (92.2%)
	≥ 37.5 °C	9 (11.4%)	9 (4.1%)	18 (6.1%)
Number of perioperative SARS-CoV-2 tests, *n* (%)				
Preoperative	1	69 (87.3%)	210 (96.8%)	279 (94.3%)
	2	5 (6.3%)	7 (3.2%)	12 (4.1%)
	3	4 (5.1%)	0	4 (1.4%)
	5	1 (1.3%)	0	1 (0.3%)
Postoperative	1	5 (6.3%)	0	5 (1.7%)
Classification of patients without confirming a negative SARS-CoV-2 result, *n* (%)				
	1	1 (1.3%)	0	1 (0.3%)
	2	3 (3.8%)	0	3 (1.0%)
	3	38 (48.1%)	0	38 (12.8%)
Treated in a negative pressure operating room, *n* (%)		42 (53.2%)	0	42 (14.2%)

Data are expressed as *n* (%). COVID-19: coronavirus disease 2019; SARS-CoV-2: severe acute respiratory syndrome coronavirus 2.

**Table 3:** Neonatal outcomes.

	Emergency (*n* = 90)	Elective (*n* = 254)	Total (*n* = 344)
Birth weight, g (SD)	2484 (900)	2922 (572)	2808 (701)
Apgar score, median (IQR)			
1 min	8 (6–9)	8 (8–9)	8 (8–9)
5 min	9 (8–9)	9 (9–9)	9 (9–9)
NICU admission, *n* (%)	43 (47.8%)	52 (20.5%)	95 (27.6%)
NICU negative pressure room admission, *n* (%)	4 (4.4%)	0	4 (1.2%)
Multifetal pregnancy case, n			
Twin	7	37	44
Triplet	2	0	2

Data are expressed as *n* (%), mean (SD), or median (IQR). SD: standard deviation; IQR: interquartile range; NICU: neonatal intensive care unit.

**Table 4:** Outcomes of the neonates isolated in the negative-pressure room.

Newborn No.	Length of Hospital Stay, Day	Length of ICU Stay, Day	Ventilation Requirements	Healthcare Infection
1 *	1	1	Yes	No
2	3	3	No	No
3	4	4	No	No
4	17	17	Yes	No

ICU: intensive care unit. * After confirmation of a negative PCR result, this newborn was transferred to another hospital due to a lack of intensive care unit.

## Data Availability

Not applicable.

## Supplementary Materials

The following are available online at https://www.mdpi.com/2077-0383/10/4/885/s1, Figure S1: COVID-19 questionnaire of our institution. COVID-19: coronavirus disease 2019.
