# Supplementary material for: Importance of Preoperative Screening Strategies for Coronavirus Disease 2019 in Patients Undergoing Cesarean Sections: A Retrospective, Large Single-Center, Observational Cohort Study
Source: J Clin Med. 2021 Feb 22;10(4):885. doi: 10.3390/jcm10040885 (PMC7926628; doi:10.3390/jcm10040885)
Supplement: Supplementary file 1 [file jcm-10-00885-s001.pdf]

COVID-19 Questionnaire

Infection control office, Asan Medical Center
2020.08.26 10:00 ver.

★★To prevent COVID-19, one designated guardian during the hospitalization period is required.
Do not allow visitors to enter the ward except for one designated guardian★★
★Please provide accurate and honest answers. Those who answer dishonestly may be penalized in accordance with the national health regulations and law.★

Patient	Name		ID		Contact number		Ward	
Guardian	Name		Relationship		Contact number			

Only High risk(★) Epidemiological Relevance Check for Outpatient & Exam

1. Epidemiological Correlation (Check All That Apply, "★" = "high-risk" category)

☐ ★**Contact with COVID-19 confirmed patient** *(2 weeks from the contact date)* *(Call 8112(888112))*
(Received notifications from national instutition after coming into contact with a patient confirmed of COVID-19 (SMS, public health center, etc.))

※ Children and adolescents under the age of 19 need to check their exposure for up to 4 weeks from the date of contact to differentiate multi-organ inflammatory syndrome.

☐ ★**Overseas visits within the last 2 weeks** (Country:) Duration(Y/M/D):__/__/__ ~ __/__/__

☐ Released from Quarantine after COVID-19 Confirmation, Quarantine Released Date(Y/M/D):__/__/__ *(Call 8112(888112))*

☐ Familial Relationship
- ○ Housemate is confirmed COVID-19
- ○ Housemate have received notifications from the government regarding contact of confirmed COVID-19
- ○ Housemate had overseas visits within the last 2 weeks

☐ Was present in an area that was visited by an COVID-19 patient [ ]

☐ ★ **Visited the following High-Risk Mass Outbreak** (Check the Visited Date and place name)
※ Not applicable if 2 weeks have passed from the last visit

Location			Visited	Name of the place	Date
Seoul	Gwanak-gu	Bongcheon-dong	☐	Youngin MC Building 6th Floor (Muhan Group)	8/10~20
	Seongbuk-gu	Jangwi-dong	☐	Sarang Jeil Church(Applies also if the housemate has visited)	7/27~8/14
	Yeongdeungpo-gu	Yeouido-dong	☐	Yeouido Full Gospel Church(Applies also if the housemate has visited)	8/16~19
	Jongno-gu	-	☐	Gwanghwamun_ Participation in Liberation Day Rally	8/15
Gyeonggi	Anyang-si	Dongan-gu	☐	Sala Days	8/14~24
Incheon	Bupyeong-gu	Cheongcheon-dong	☐	Galilee Presbyterian Church(Applies also if the housemate has visited)	8/16~23
	Seo-gu	Simgok-dong	☐	Church of the Lord(Applies also if the housemate has visited)	8/16~18
Chungnam	Cheonan-si	Dongnam-gu	☐	Soon Chung Hyang University Cheonan Hospital (Excluding COVID-19 Screening Center)	8/10~24
Jeonnam	Suncheon-si	Deokwol-dong	☐	Cheongam Fitness & Spa (Gym inside Cheongam College)	8/18~21

☐ **Visited the following places (Check the Visited Date and place name)**
※ Not applicable if 2 weeks have passed from the last visit

Location			Visited	Name of the place	Date
Seoul	Gangdong-gu	Gil-dong	☐	★ Gangdong Heart Hospital Operating Room	8/16~21
	Gwangjin-gu	Jayang-dong	☐	★ Green Pastures Community Church	8/16~22
	Geumcheon-gu	Siheung-dong	☐	★ Hee Myoung Hospital (Excluding COVID-19 Screening Center)	8/20~24
	Seongdong-gu	Haengdang-dong	☐	★ Hanyang University Medical Center Hemodialysis Center	8/20~24
	Eunpyeong-gu	Bulgwang-dong	☐	★ Haircock Yeonshinnae Branch	8/8~22
	Jungnang-gu	Myeonmok-dong	☐	★ Green Hospital(Excluding COVID-19 Screening Center)	8/16~24
		Muk 1-dong	☐	★ Haesung Church	8/21~22
Gyeonggi	Gunpo-si	Dang-dong	☐	★ Fortune Restaurant	8/19~23
	Namyangju-si	Dasan-dong	☐	Gyeongcheon Yein Oriental Medical Clinic	8/20~21
		Byeolnae-dong	☐	365 DK Clinic	8/17~21
	Seongnam-si	Bundang-gu	☐	Cha University Bundang Medical Center Emergency Room, Cancer Center(Excluding COVID-19 Screening Center)	8/20~22
	Bucheon-si	Simgokbon-dong	☐	★ Haedoc Jjimjil Café	8/19~25

Location			Visited	Name of the place	Date
Gyeonggi	Pyeongtaek-si	Hyeonhwa-ri	☐	★ Lee Jong In Language Academy	8/19~21
	Hanam-si\	Deokpung-dong	☐	★ Wellbeing Bath (Jjimjilbang & Men Bath)	8/21~23
Incheon	Gyeyang-gu	Jakjeon-dong	☐	★ Duri Obstetrics and Gynecology Internal Medicine Clinic	8/21~24
	Michuhol-gu	Hakik-dong	☐	★ Michuhol Elderly Day Care Center	8/19~23
Gangwon	Wonju-si	Dangye-dong	☐	★ Popcorn PC Bang	8/21
		Dangu-dong	☐	★ Kyung Hee Hyo Day Care Center	8/19~24
			☐	★ Joy Club Multi Gym	8/20~21
		Ilsan-dong	☐	★ Wonju Severance Christian Hospital(Excluding COVID-19 Screening Center)	8/23~24
			☐	★ Wonju Green Hospital(Excluding COVID-19 Screening Center)	8/21~24
Chungbuk	Chungju-si	Cheongwon-gu	☐	★ Cheongju St. Mary's Hospital	8/21
	Chungju-si	Cheongwon-gu	☐	★ Cheongju St. Mary's Hospital(Excluding COVID-19 Screening Center)	8/20~21
Daejeon	Yuseong-gu	Bongmyeong-dong	☐	★ Yuseong Hot Spring Bulgama Sauna	8/15~21
			☐	★ Lee Boyoung's Talking Club Doan Yuseong Branch	8/21
	Jung-gu	Seonhwa-dong	☐	★ Swimming pool in Jung-gu National Sports Center	8/21
Gwangju	Seo-gu	Nongseong-dong	☐	★ Woongjin Thinkbig (Jeonnam Business Headquarters)	8/17~21
Jeonnam	Suncheon-si	Gagok-dong	☐	★ Yusimcheon	8/21
		Deokam-dong	☐	★ Best Hospital(Excluding COVID-19 Screening Center)	8/18~22
		Deokwol-dong	☐	★ Awesome PC Café	8/19, 21
		Dongoe-dong	☐	★ Han Gajok Full Gospel Church	8/22
		Jorye-dong	☐	★ Kim Seonsaeng Fitness Club	8/18~21
Gyeongnam	Geoje-si	Geoje-myeon	☐	★ Busong Noodles	8/14~22
Busan	Jin-gu	Gaya-dong	☐	★ Gaya Spa Valley 24 (Women's Bath)	8/16~22
Jeju	Jeju-si	Hwabukil-dong	☐	★ Hwabuk Cathedral	8/23

☐ None of the Above(Epidemiological Correlation)

2. Check All Symptoms That Apply(Symptoms that occured or are getting worse within 2 weeks)

* Please put a check if there are symptoms within 2 weeks even if there are no current symptoms

☐ Fever above 37.5℃ [Took Fever Reducer Y or N, Date and Time of Last Intake:]

(If you have taken a fever reducer within 6 hours, you will be considered to have a fever even if you don't actually have a fever)

☐ Cough ☐ Muscle ache (Body Aches) ☐ Chill

☐ Headache ☐ Smell or taste loss ☐ Sore Throat

☐ New symptoms of nasal congestion(New symptom of nasal congestion or runny nose for Children´s Hospital)

☐ Difficulty Breathing ☐ Pneumonia ☐ None of the Above

3. Additional checklist(**Only for** inpatient and emergency patients)

☐ (Within 2 weeks) Confirmation regarding Nursing Facilities

○ Admitted in Nursing Facilities or Received Day Care Center Service

○ Received Home-Visit Care Service from Care Worker (Excluding Home Healthcare)

○ Working as a Care Worker

(Facility name:), Duration(Y/M/D): ___/___/___ ~ ___/___/___

→ **If applicable, follow up for SARS-CoV-2 will be done 3 days after admission [HD#4]**

☐ Lives in **Seoul, Gyeonggi, and Incheon**

→ If applicable, SARS-CoV-2 exam will be done **a day before hospitalization**. After checking negative results, the hospitalization will proceed the following day

★ If the information is different from what you have written, assigned hospital bed can be changed and admission procedure may be delayed. Additional examination may be also required so please fill out the information correctly. ★

I confirm that the information provided above is accurate to best of my knowledge.

Date signed (Y/M/D): 2020 / ____ / ____

Print Name and Sign : (signature)

Medical Staff : (signature)

Supplementary Figure S1. COVID-19 questionnaire of our institution
